# Reliability of a patient survey assessing cost-related changes in health care use among high deductible health plan enrollees

**DOI:** 10.1186/1472-6963-11-133

**Published:** 2011-05-27

**Authors:** Robert B Penfold, Jeffrey T Kullgren, Irina Miroshnik, Alison A Galbraith, Virginia L Hinrichsen, Tracy A Lieu

**Affiliations:** 1 Department of Population Medicine, Harvard Medical School and Harvard Pilgrim Health Care Institute, Boston, USA; 2 Group Health Research Institute, 1730 Minor Avenue, Suite 1600, Seattle, WA 98101, USA; 3 Robert Wood Johnson Clinical Scholars, Philadelphia Veterans Affairs Medical Center and University of Pennsylvania, Philadelphia, USA; 4 Leonard Davis Institute of Health Economics, University of Pennsylvania. Philadelphia, USA; 5 Division of General Pediatrics, Children's Hospital Boston, Boston, USA

## Abstract

**Background:**

Recent increases in patient cost-sharing for health care have lent increasing importance to monitoring cost-related changes in health care use. Despite the widespread use of survey questions to measure changes in health care use and related behaviors, scant data exists on the reliability of such questions.

**Methods:**

We administered a cross-sectional survey to a stratified random sample of families in a New England health plan's high deductible health plan (HDHP) with ≥ $500 in annualized out-of-pocket expenditures. Enrollees were asked about their knowledge of their plan, information seeking, behavior change associated with having a deductible, experience of delay in care due in part to cost, and hypothetical delay in care due in part to cost. Initial respondents were mailed a follow-up survey within two weeks of each family returning the original survey. We computed several agreement statistics to measure the test-retest reliability for select questions. We also conducted continuity adjusted chi-square, and McNemar tests in both the original and follow-up samples to measure the degree to which our results could be reproduced. Analyses were stratified by self-reported income.

**Results:**

The test-retest reliability was moderate for the majority of questions (0.41 - 0.60) and the level of test-retest reliability did not differ substantially across each of the broader domains of questions. The observed proportions of respondents with delayed or foregone pediatric, adult, or any family care were similar when comparing the original and follow-up surveys. In the original survey, respondents in the lower-income group were more likely to delay or forego pediatric care, adult care, or any family care. All of the tests comparing income groups in the follow-up survey produced the same result as in the original survey.

**Conclusions:**

In this population of HDHP beneficiaries, we found that survey questions concerning plan knowledge, information seeking, and delayed or foregone care were moderately reliable. Our results offer reassurance for researchers using survey information to study the effects cost sharing on health care utilization.

## Background

Increases in health care costs have led to increases in patient cost-sharing arrangements such as high deductibles. The percentage of Americans insured by high deductible health plans (HDHPs) - also known as consumer-directed health plans (CDHPs) when combined with a health savings account or health reimbursement account - increased from 8% in 2006 to 17% in 2009[1,2]. The effects of cost-sharing arrangements typically have been evaluated in two ways: through analyses of claims data, and through patient surveys that ask about changes in health care decision making and use.

Some of the most important data on the effects of increases in cost-sharing for health care in the United States come from surveys [3-5]. Reed and colleagues[5], for example, asked respondents how often they changed their care-seeking behavior because of their out-of-pocket costs. The investigators compared self-reported and claims-based cost-sharing levels to assess consumers' knowledge of their cost-sharing plan. Fronstin[2] also asked health plan enrollees about changes in care seeking. He found that CDHP enrollees were more likely than traditional plan enrollees to report that they would change doctors if cost sharing was lower when using a doctor who used health information technology. Collins and colleagues asked if, because of cost, respondents did not go to a doctor or clinic when sick; had not filled a prescription; skipped a medical test, treatment, or follow-up visit recommended by a doctor; or did not see a specialist when a doctor or the respondent thought it was needed [6]. The proportion of individuals forgoing these types of care increased from 29 percent in 2001 to 45 percent in 2007 [6].

Despite the widespread use of survey questions to measure changes in health care use and related behaviors, scant data exists on the reliability of such questions. If these questions do not produce reliable responses, this would blunt the ability of studies to identify true changes in health care use associated with different health insurance cost-sharing arrangements. A better understanding of the reliability of patients' responses to questions regarding their experiences delaying care and/or hypothetical health care seeking behavior would improve our ability to predict cost-related changes in health care use.

We sought to measure the reliability of survey questions assessing changes in health care utilization and delayed or foregone care among a group of enrollees in HDHPs. While there is a vast body of literature reporting on the reliability and validity of various psychometric instruments [7-10], and health related quality of life questionnaires [11-13], similar work has not been done regarding delayed or foregone health care due in part to cost. To our knowledge, this is the first study to examine the test-retest reliability of questions regarding self-reported information use, health care utilization, and delayed or foregone care among HDHP enrollees using a survey instrument administered at two points in time to the same individuals.

In addition to evaluating the test-retest reliability of select survey questions, we also report aggregate differences in information use, health care utilization and delayed or foregone care in order to evaluate how potential changes, due in part to unreliability (which may be related to question design, survey administration, patient recall, the time interval between responses, data entry, etc), might affect the findings of an analysis using a single cross-section of survey data.

## Methods

### Study Population

The study population consisted of enrollees in HDHPs offered by Harvard Pilgrim Health Care, a New England-based non-profit health insurer. A description of the population and plan characteristics has been previously published [14]. The target sample included adults 18 years of age and older who, as of November 2008, were subscribers enrolled in a Harvard Pilgrim Health Care HDHP with an individual deductible of at least $1000 and a family deductible of at least $2000. All non-preventive inpatient, outpatient and emergency department care was subject to the deductible under these plans. Some preventive care was exempt from the deductible and only subject to a minimal copayment. Some diagnostic testing is also exempt from deductibles (e.g., fecal occult blood tests are exempt but colonoscopy is not). We focused our survey questions regarding hypothetical utilization on diagnostic tests for which the deductible applies (blood tests, colonoscopy, magnetic resonance imaging).

The inclusion criteria also required: (1) continuous enrollment in an employer-sponsored HDHP for at least the previous 6 months; (2) at least one child < 18 years of age also enrolled in the plan; and (3) annualized family out-of-pocket costs (defined as outpatient visit and prescription drug co-pays) of at least $500 in an HDHP. This threshold of annualized OOP expenses included 54% of all families who met other inclusion criteria. We required at least $500 in OOP expenses to ensure that the questions concerning delayed or forgone care would be relevant to potential respondents. We reasoned that individuals who consumed some care would have some experience with their own plan and recent decisions to delay or forego care, and be more able to readily judge hypothetical decision making at different cost levels.

We oversampled households living in low-income areas by stratifying families into two groups based on geocoded address information: (1) residence in a census block group with a median household income in the lowest quartile in the sample frame and (2) residence in a census block group with a median household income in the second, third and fourth quartiles of the sample frame (i.e., lowest quartile *versus *other). Low income individuals were oversampled because nominal increases in cost sharing affect this population of enrollees disproportionately to their income; thus, these enrollees may be more likely to delay or forego care due to cost. Random sampling was performed in each of the two strata until surveys from approximately 200 families in each group were completed.

Analyses were stratified by self-reported household income on the survey. The lower-income subgroup was defined by a self-reported household income less than 300 percent of the Federal Poverty Limit (FPL < 300). Families with incomes above this threshold were classified as higher-income.

### Survey Design

The survey included several domains: health plan characteristics, attitudes towards health care utilization, unexpected costs, information-seeking behaviors, delayed care, and demographic data. Survey domains and questions were developed based on a previous focus group study in this population and were in some cases drawn from existing national surveys [15]. The draft survey underwent cognitive pre-testing and piloting with a total of 60 respondents. The study was approved by the Harvard Pilgrim Health Care Institutional Review Board.

### Survey Dissemination and Re-sampling

The original (i.e. the "test") surveys were mailed between January and March 2009. The cover letter asked the adult in the family who is responsible for the family's health care decisions to complete the survey. We sent two mail waves followed by attempts at telephone administration. Respondents received a $30 gift card to their choice of 1 of 3 major retailers. Of the 750 surveys mailed, 229 (30.5%) were completed in the first wave, 130 (17.3%) were completed in the second wave, and 75 (10%) were completed by phone for a total of 434 completed surveys and an overall response rate of 58.1%.

We attempted to administer the follow-up (i.e., the "retest") survey to all 434 families who completed the original survey. The follow-up survey cover letter explained that, "the reason we are asking you to do these questions a second time is to test how reliable the questions are - whether they result in the same answers or different answers if asked at different times."

Follow-up surveys were distributed (mail) or administered (telephone) within two weeks of each family responding to the original survey; however, individual response time varied. The follow-up surveys were all completed between February and July of 2009. We attempted the survey in the same format in which the respondents completed the original survey (i.e., mail or phone), using the same incentives and number of mailing waves. The questions were identical between the original and follow-up surveys. Five questions regarding demographic information (race, ethnicity, language spoken at home, education, income) and two administrative questions (suggestions for improvement, permission to obtain administrative claims data) were omitted from the follow-up survey.

### Survey Themes and Questions

**Figure 1:**
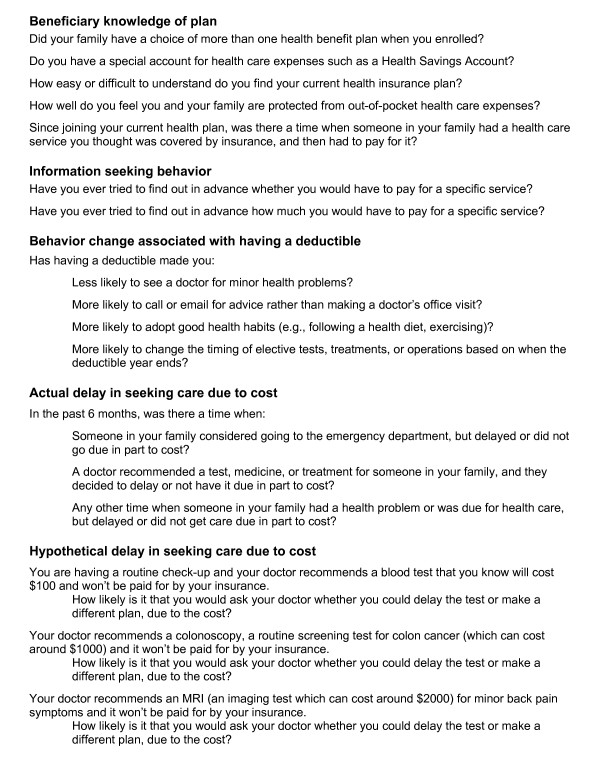
**Test-retest questions**.

The survey covered five broad themes: beneficiary knowledge of their health plan, information seeking, changes in behavior associated with having a deductible, experiences in delayed or foregone care due in part to cost, and hypothetical delays in care due in part to cost. Figure 1 contains the 17 questions tested for reliability under each theme.

### Statistical Analysis

Our statistical analysis involved 3 components. We compared the agreement of (1) individual level responses between the original and follow-up surveys via percent agreement, absolute retraction, absolute initiation, tetrachoric correlation (binary responses) or polychoric correlation (ordinal categorical responses), and kappa statistics; (2) differences in responses between income groups - comparing the original versus follow-up responses; and (3) differences in responses between the original and follow-up responses, stratified by income group.

We calculated the percentage agreement [16] for each of the 17 questions in order to capture the proportion of respondents that gave identical answers on the original and follow-up surveys. We also calculated absolute retraction and initiation. Absolute retraction is the proportion of individuals who responded positively to a behavior on the original survey (e.g., responded yes to having a health savings account) and responded negatively to the behavior on the follow-up survey. Conversely, absolute initiation measures the proportion of respondents that responded negatively on the original survey and positively on the follow-up survey (e.g., unlikely to delay care on the original and likely to delay care on follow-up). For questions involving ordinal responses, absolute retraction and initiation were calculated by summing the off-diagonal counts and dividing by the total responses. In the case of absolute retraction, this method implies any reduction in the level of endorsement (e.g., very likely changed to somewhat likely, somewhat unlikely, or very unlikely). Similarly, absolute initiation implies any increase in the level of endorsement (very unlikely changed to somewhat unlikely, somewhat likely, or very likely).

We also calculated simple (for binary response) and weighted (for ordinal response) kappa statistics for each question [17,18]. We used Landis and Koch's standard criteria for interpreting the strength of kappa which are: 0.0 - 0.2 (slight); 0.21 - 0.40 (fair); 0.41 - 0.60 (moderate); 0.61 - 0.80 (substantial); and 0.81 1.0 (almost perfect) [19,20]. While kappa is the most common statistic used to compare categorical survey responses at two points in time and has been used to evaluate national surveys in the United States [21], kappa has several weaknesses including sensitivity to the prevalence of a behavior/response [22], the number of categories, and the assumption of independent raters [23-28]. We therefore calculated tetrachoric correlations (TCC) and polychoric correlations (PCC) to provide measures of agreement for binary response questions (TCC) and ordinal categorical response questions (PCC) between the original and follow-up surveys. Both the TCC and PCC are independent of prevalence so low agreement cannot be attributed to low prevalence or a change in prevalence between survey waves [29-32].

In a previous study [14], we used χ^2 ^tests to investigate whether lower-income families with out-of-pocket expenditures in HDHPs were more likely than higher-income families to delay or forego health care services, have difficulties understanding and using their plans, or avoid discussing costly services with physicians. We repeated these analyses for the present study but restricted the original sample to individuals that responded to the follow-up survey in order to evaluate the extent to which our initial findings would be reproduced.

We compared the responses in the lower-income group (FPL < 300%) between the original and follow-up surveys using *McNemar's test *[33,34]. The same analysis was used to compare responses in the higher-income group (FPL ≥ 300%) between the original and follow-up surveys.

All statistical analyses were performed using SAS version 9.2 [35].

## Results

Of the 434 original completed surveys, 387 completed the follow-up survey (310 written, 77 by phone) and 47 did not respond giving a response rate of 89%. Twenty-four (6%) of the 387 follow-up surveys were completed in a different format than the original survey (23 of 24 were completed by phone in the follow-up and on paper in the original survey; 1 survey was completed on paper in the follow-up and by phone in the original).

Although follow-up surveys were fielded within two weeks of receiving the initial survey, time between receipt of the initial survey and receipt of the follow-up survey varied. The mean time between initial and follow-up survey receipt was 29.7 days (95% CI: 28.3, 31.0) and the median time was 26 days. Of the 387 follow-up surveys, 75% were returned within 31 days and 95% were returned within 56 days. A little less than 5% (n = 19) of follow-up surveys were returned between 57 and 142 days after the initial survey. Several studies have reported reliable estimates of retrospective and contemporaneous behaviors in a 2 to 10 week test-retest period [8,9,36-38].

We tested for differences in characteristics between follow-up survey respondents and non-respondents such as age, sex, minority status, education, family size, income, deductible level, enrollment time, choice of plan, and chronic illness among family members. The initial and follow-up surveys differed only with respect to the proportion of respondents with any adult chronic condition in the family (including allergies). Of the 47 non-respondents, 37 (78.7%) had an adult in the family with a chronic condition compared to 57.8% of initial respondents (χ^2 ^p = 0.028).

### Question Test-Retest Reliability

Table 1 summarizes the agreement statistics describing the reproducibility of respondents' answers on the original and follow-up surveys. The question on whether the respondent had a special account for health care expenses such as a health savings account had the highest test-retest reliability, with a percent agreement of 93%, TCC of 0.97 (95% CI: 0.95, 0.99) and kappa of 0.84 (95% CI: 0.78, 0.90). The other items concerning beneficiary knowledge of their plan had moderate kappa values. Although the test-retest kappa statistic concerning choice of plans was 0.61 (substantial), the confidence interval for kappa (0.52, 0.69) overlapped that of the items concerning the ease/difficulty of understanding their plan (95% CI: 0.41, 0.56) and how protected the beneficiaries feel from out-of-pocket costs (95% CI: 0.43, 0.57). Note that these confidence intervals are based on the assumption of independent raters; however, the individual answers for the original and follow-up surveys are clearly dependent. As such, the confidence intervals are conservative (wide) and favor finding no difference between kappa values. Use of overlapping confidence intervals is generally conservative compared to standard error intervals [39,40]. Nevertheless, the magnitude of the differences in kappa is small compared to the Landis and Koch [19] criteria (0.41 - 0.60 = moderate); thus, comparisons via narrower confidence intervals might be statistically significant but unimportant when falling within the same categorical band (i.e., moderate).

**Table 1:** Agreement statistics for the families and health care costs survey: comparison of responses between the original and follow-up surveys

Survey Domain	Percent	Absolute	Absolute	TCC or	95% C.I.			95% C.I.	
	Agree	Retraction	Initiation	PCC	CC-L	CC-U	κ	κ-L	κ-U
**Beneficiary Knowledge of Plan**									
Choice of more than one health plan?	80.6%	10.2%	9.2%	0.82	0.74	0.89	0.61	0.52	0.69
Health Savings Account?	92.9%	4.0%	3.2%	0.97	0.95	0.99	0.84	0.78	0.9
Ease/difficulty of understanding plan?	65.8%	18.5%	15.7%	0.72	0.65	0.79	0.48	0.41	0.56
How protected from expenses?	59.2%	20.0%	20.8%	0.70	0.63	0.76	0.50	0.43	0.57
**Information Seeking Behavior**									
Tried to discover whether required to pay?	75.9%	11.4%	12.7%	0.73	0.63	0.82	0.51	0.42	0.6
Tried to discover how much required to pay?	76.2%	10.6%	13.2%	0.72	0.63	0.82	0.52	0.43	0.6
**Behavior Change Associated with a Deductible**									
Less likely to see doctor?	76.9%	8.7%	14.4%	0.75	0.66	0.84	0.53	0.43	0.61
More likely to call/email and not visit?	71.0%	11.2%	17.8%	0.62	0.50	0.73	0.41	0.32	0.51
More likely to adopt good health habits?	71.7%	12.4%	15.9%	0.63	0.52	0.74	0.43	0.34	0.53
Deductible: more likely to change timing?	75.6%	11.4%	13.0%	0.67	0.57	0.78	0.46	0.37	0.55
Any behavior change?*	84.8%	6.7%	8.5%	0.71	0.59	0.83	0.45	0.33	0.57
**Experienced Delay in Care Due in Part to Cost**									
Delayed or forgone ED visit?	87.4%	6.5%	6.0%	0.88	0.82	0.94	0.67	0.58	0.75
Delayed or foregone recommended test?	80.6%	6.6%	12.9%	0.76	0.66	0.85	0.52	0.42	0.61
Delayed/foregone any other time?	82.9%	9.6%	7.5%	0.70	0.58	0.82	0.45	0.34	0.56
Any delayed or foregone care (ED, test, other)?*	81.4%	9.8%	8.8%	0.83	0.76	0.90	0.62	0.55	0.7
**Hypothetical Delay in Care Due in Part to Cost**									
$100 Blood test - Likelihood of delay	52.7%	30.0%	17.2%	0.63	0.56	0.71	0.45	0.38	0.52
$1000 Colonoscopy - Likelihood of delay	63.4%	23.9%	12.7%	0.74	0.67	0.80	0.53	0.46	0.6
$2000 MRI - Likelihood of delay	74.6%	16.1%	9.3%	0.75	0.67	0.82	0.53	0.44	0.61

* Composite measure of all individuals answering yes to at least one behavior change or delayed/forgone care.

The results comparing agreement via the TCC or PCC are similar to those using kappa. While the level of agreement reported for each question is generally higher using TCC/PCC, the confidence intervals for these estimates overlap except with respect to having a health savings account. The reliability of the question regarding delays in ED care (PCC = 0.88, 95% CI: 0.82, 0.94) may also be higher than that for delayed or foregone care at "any other time" (PCC = 0.70, 95% CI: 0.58, 0.82).

The test-retest reliability as measured by kappa was moderate for the majority of questions (0.41 - 0.60). Similarly, 14 of the 18 correlations (TCC/PCC) are between 0.62 and 0.76 which could also be interpreted as moderate agreement. Interestingly, the level of test-retest reliability for questions concerning the experience of delays in care did not differ substantially from questions regarding hypothetical delays in care. The kappa statistic for delayed or foregone emergency department care was 0.67 (95% CI: 0.58, 0.75), significantly higher than most questions related to behavior change associated with having a deductible (i.e., more likely to call/email, more likely to adopt healthy habits, more likely to change the timing of visits). These results remain statistically significant using either kappa or the TCC/PCC.

### Reproducibility of Comparisons Regarding Information Seeking and Delayed or Foregone Care

Table 2 shows the results of bivariate analyses comparing income groups based on responses to the original and the follow-up survey. The observed proportions of respondents with delayed or foregone pediatric, adult, or any family care were similar when comparing the original and follow-up surveys. All of the tests comparing income groups in the follow-up survey produced the same result as in the original survey: respondents in the lower-income group were more likely to delay or forego pediatric care, adult care, or any family care in both surveys.

**Table 2:** Comparison of delayed or foregone care between federal poverty limit < 300 and federal poverty limit >300 in the original and follow-up surveys

	Original (n = 387)†			Follow-up (n = 387)			*P *values ‡	
Type of Care	FPL < 300%	FPL ≥ 300%	*P *value* Original Survey	FPL < 300%	FPL ≥ 300%	*P *value* Follow-up Survey	FPL < 300 Between Original and Follow-up	FPL ≥ 300 Between Original and Follow-up
**Any Delayed or Foregone Care**								
Pediatric care	24.0	13.2	0.014	24.0	10.8	0.002	1.000	0.439
Adult care	48.8	33.6	0.007	51.2	32.8	0.001	0.785	0.877
Family care	56.2	41.6	0.011	58.7	38.8	0.001	0.799	0.621
**Types of delayed or foregone care**								
ED visit	61.8	52.9	0.322	**69.0**	**45.4**	**0.004**	0.463	0.269
Preventive care	27.9	18.3	0.191	25.4	18.6	0.384	0.869	0.869
Imaging test	27.9	23.1	0.589	31.0	25.8	0.569	0.639	0.886
Prescription medication	17.7	**15.4**	0.856	12.7	**5.2**	0.144	0.513	**0.016**
Specialist visit	19.1	12.5	0.334	18.3	13.4	0.514	1.000	1.000
Screening test	17.7	11.5	0.365	18.3	10.3	0.207	0.841	0.670
Laboratory test	14.7	8.7	0.323	19.7	10.3	0.134	0.414	0.819
Operation or procedure	**19.1**	**5.8**	**0.013**	18.3	11.3	0.293	1.000	0.225
Physical therapy	11.8	12.5	1.000	14.1	11.3	0.768	0.637	0.683
Outpatient visit for routine check-up	10.3	6.7	0.582	9.9	8.25	0.93	1.000	0.796

The p-values in columns 3 and 6 represent the observed type-1 error (α) for tests comparing income groups whereas the p-values in columns 7 and 8 are the observed α for tests comparing the original and follow-up surveys.

*P values are for chi-square tests between income groups within survey

† We restricted the original survey data to follow-up respondents

‡ P values are for McNemar tests within income groups and between surveys

Bolded items signify comparisons that are statistically significant at α = 0.05

The results of difference of proportion tests comparing income groups are also largely similar. However, in two cases there were significant differences. The test comparing delayed or foregone ED care was statistically significant in the follow-up survey but not in the original survey. Also, the test comparing delayed or foregone operations/procedures was statistically significant in the original survey but not in the follow-up survey.

Table 2 also shows the results of our analyses comparing the proportion of respondents with delayed or foregone care between the original and follow-up surveys, stratified by income level. With one exception, none of the difference of proportion tests comparing the original and follow-up surveys within income groups was statistically significant. The proportion of respondents in the higher-income group that delayed or went without prescription medications decreased from 15.4 percent in the original survey to 5.2 percent in the follow-up survey (p = 0.016).

Table 3 shows the results of analyses comparing the proportion of respondents who answered that their plan was difficult to understand, that they had unexpected costs, that they felt unprotected from out-of-pocket (OOP) expenses, and the types of information seeking they conducted (i.e., whether required to pay and/or how much they were required to pay). In both the original and follow-up surveys, lower-income respondents were more likely to report that they felt unprotected from OOP expenses.

**Table 3:** Comparison of information seeking between federal poverty limit <300 and federal poverty limit >300 in the original and follow-up surveys (%)

	Original (n = 387)†			Follow-up (n = 387)			*P *values‡	
Plan use and information-seeking	FPL < 300%	FPL ≥ 300%	*P *value*	FPL < 300%	FPL ≥ 300%	*P *value*	FPL < 300 Between Original and Follow-up	FPL ≥ 300 Between Original and Follow-up
Unprotected from OOP expenses	55.8	44.0	0.043	60.8	40.2	<0.001	0.553	0.577
Plan difficult to understand	24.8	26.1	0.886	30.8	25.0	0.290	0.392	0.859
Unexpected costs since joining plan	46.2	41.2	0.425	47.1	49.2	0.789	0.269	0.183
Am I required to pay?	50.0	51.6	0.860	50.4	53.2	0.695	1.000	0.439
How much am I required to pay?	40.8	39.8	0.945	43.3	41.7	0.854	0.841	0.722

The p-values in columns 3 and 6 represent the observed type-1 error (α) for tests comparing income groups whereas the p-values in columns 7 and 8 are the observed α for tests comparing the original and follow-up surveys.

*P values are for chi-square tests between income groups within survey

† We restricted the original survey data to follow-up respondents

‡ P values are for McNemar tests within income groups and between surveys

In the original survey, 55.8% of respondents in the lower-income group reported feeling unprotected from OOP expenses versus 44.0% of those in the higher-income group (p = 0.043). The difference between income groups widened in the follow-up survey where 60.8% of lower-income respondents reported feeling unprotected versus 40.2% in the higher-income group (p < 0.0001). None of the other income group comparisons were significant and none of the proportions changed significantly between the original and follow-up surveys.

## Discussion

Although surveys are widely used to measure self-reported and hypothetical use of health care by enrollees with cost sharing arrangements, the test-retest reliability of these surveys has not been adequately studied. A better understanding of the reliability of patients' responses to questions on health care seeking behavior is important for improving our ability to identify true changes in health care use associated with different health insurance cost-sharing arrangements.

The test-retest reliability of self-reported plan knowledge, information seeking, and delayed or foregone care reported in this study can generally be characterized as "moderate". None of the questions with kappa statistics in the "substantial" range had confidence intervals that were completely within the substantial range. The apparent consistency of kappa statistics across the various domains is interesting because respondents were equally reliable answering questions about their experiences in delaying care as they were answering questions about hypothetical delays in care. However, readers should exercise care in interpreting differences in these kappa as the confidence intervals are conservatively wide.

We found that most of the proportions of respondents reporting delayed or foregone care did not change significantly between the original and follow-up surveys. Only the proportion of higher-income respondents reporting delayed or foregone prescriptions medications changed. In comparing the lower-income and higher-income groups, only the results concerning delayed or foregone ED care and delayed or foregone operations/procedures changed between the original and follow-up surveys. These results suggest that we can be reasonably confident in our initial analyses and the propensity to delay or forego care in this population of beneficiaries.

### Plan Use and Information Seeking

Results across the original and follow-up surveys show that 40 to 50 percent of respondents experienced unexpected costs or felt unprotected from OOP costs. Similarly, only 40 to 50 percent of respondents reported trying to discover whether or not they would have to pay for a service and/or how much they would have to pay for a service since joining their health plan. Although the proportions describing plan use and information seeking in each income group changed slightly between the original and follow-up surveys, none of these changes were statistically significant. Our analysis of the follow-up sample confirmed the results reported for the original study: a higher proportion of lower-income individuals reported feeling unprotected from OOP expenses. We also confirmed our previous analysis in that none of the other comprehension/information proportions were different in the lower-income group compared to the higher income group. These results suggest that we can be confident in our initial results comparing plan knowledge and information seeking.

### Strengths and Limitations

This study has two main strengths. This is the first test-retest reliability study of questions concerning beneficiaries' self-reported understanding and use of their HDHP benefits. Second, we repeated the analyses comparing lower-income and higher-income individuals in addition to calculating a variety of agreement statistics for the individual questions. Repeating the analyses by income allowed us to determine whether there were significant changes in the proportion of people reporting delayed or foregone care between the original and follow-up surveys. We found that we can be confident in our initial results using the original survey data because only one proportion out of eighteen changed despite the moderate level of reliability as captured by the agreement statistics.

One limitation of our study is that the results may be biased by individuals' recall of their initial responses when completing the follow-up survey. Given the moderate level of test-retest reliability, the learning effect does not appear to have been strong. However, the absolute initiation among respondents with respect to experienced delays (Table 1, Behavior Change) is consistently greater than the absolute retraction. Another limitation is that we do not know the extent to which respondents provided different answers due to events that occurred in the period between completing the original and follow-up surveys. Almost 5% (n = 19) of follow-up surveys were returned between 57 and 142 days (approximately 2 - 5 months) after the initial survey; thus, the possibility that new health care utilization affected follow-up survey responses cannot be ignored. On the other hand, several studies have reported reliable estimates of retrospective and contemporaneous behaviors in a 2 to 10 week test-retest period [8,9,36-38].

These data may not be representative of all HDHP populations because our sample was limited to enrollees in one New England health plan. We focused on families who had experienced high costs, who may have experienced more salient events than others, or have been more likely to recall events reliably. Survey questions regarding hypothetical utilization centered on diagnostic tests for which the deductible applies; however, confusion over which diagnostic services are subject to the deductible could be an important source of variability in patient responses to experiences in delayed or foregone care between the initial and follow-up surveys. Further, our inclusion criterion of $500 in annualized visit and prescription drug co-payments may have excluded families who faced access barriers so significant that they never reached this level of out-of-pocket costs, and limits our ability to generalize these findings to individuals with either much lower or much higher out-of-pocket spending. The reliability of the survey instrument for HDHP enrollees with no OOP expenses is likely to be lower because they would have little or no experience with their plan and recent decision making. As such, the reliability estimates presented here may be optimistic for a survey fielded to all HDHP enrollees regardless of their plan experience.

As in other studies of HDHPs, families who were enrolled in these plans may differ in important and often unobservable ways from those who were not, whether they actively chose the plan or had no other option. Our measures gauging respondents' willingness to discuss hypothetical recommended services also may not be predictive of their actual behavior, although we found similar reliability for questions regarding experienced and hypothetical delays in care. Finally, the lack of a non-HDHP comparison group limits the degree to which our observed income group differences and similarities can be contrasted with health plans that have small or no deductibles.

## Conclusions

In this population of HDHP beneficiaries, we found that self-reported information concerning plan knowledge, information seeking, and delayed or foregone care was moderately reliable. Our results offer reassurance for researchers using self-reported information to study the effects of changes in cost sharing on health care utilization.

Payers and policy makers are increasingly interested in benefit structures that maximize the use of appropriate health care and minimize the use of inappropriate care. The results presented here complement studies using retrospective administrative data to evaluate changes in the use of health care associated with deductible levels. Our results suggest that beneficiary surveys concerning hypothetical changes in deductibles could be reliably used to better understand potential changes in utilization that might occur under different deductible levels and plan designs (i.e., services subject to and exempt from the deductible). As the proportion of Americans with cost-sharing arrangements for health care continues to increase, reliable self-reported information about their health care decision processes and use will become increasingly important.

## Competing interests

The authors declare that they have no competing interests.

## Authors' contributions

RP contributed to data analysis and wrote the manuscript. JK contributed to data analysis and interpretation and the writing of the manuscript. IM contributed to the design and conduct of the data analysis. AG contributed to data analysis and interpretation and the writing of the manuscript. VH collected the data and critically read the manuscript. TL designed the study, arranged funding, contributed to data analysis and interpretation, and critically revised the manuscript for intellectual content. All authors approved the final version of the manuscript.

## Pre-publication history

The pre-publication history for this paper can be accessed here:

http://www.biomedcentral.com/1472-6963/11/133/prepub

## References

[B1] Cohen RA Martinez ME Consumer-directed health care for persons under 65 years of age with private health insurance: United States, 2007 NCHS Data Brief 2009 1 8 19389327

[B2] Fronstin P Findings from the 2009 EBRI/MGA Consumer Engagement in Health Care Survey EBRI Issue Brief 2009 1 42 20043408

[B3] Dixon A Greene J Hibbard J Do consumer-directed health plans drive change in enrollees' health care behavior? Health Aff (Millwood) 2008 27 1120 1131 10.1377/hlthaff.27.4.1120 18607046

[B4] Reed M Benedetti N Brand R Newhouse JP Hsu J Perspectives from deductible plan enrollees: plan knowledge and anticipated care-seeking changes BMC Health Serv Res 2009 9 244 10.1186/1472-6963-9-244 20040076 PMC2811111

[B5] Reed M Fung V Price M Brand R Benedetti N Derose SF Newhouse JP Hsu J High-deductible health insurance plans: efforts to sharpen a blunt instrument Health Aff (Millwood) 2009 28 1145 1154 10.1377/hlthaff.28.4.1145 19597214

[B6] Losing Ground: How the Loss of Adequate Health Insurance is Burdening Working Families. Findings from the Commonwealth Fund Biennial Health Insurance Surveys 2001 http://www.commonwealthfund.org/~/media/Files/Publications/Fund%20Report/2008/Aug/Losing%20Ground%20%20How%20the%20Loss%20of%20Adequate%20Health%20Insurance%20Is%20Burdening%20Working%20Families%20%208212%20Finding/Collins_losinggroundbiennialsurvey2007_1163%20pdf.pdf

[B7] Gentile DA Woodhouse J Lynch P Maier J McJunkin T Reliability and validity of the Global Pain Scale with chronic pain sufferers Pain Physician 2011 14 61 70 21267043

[B8] Milton K Bull FC Bauman A Reliability and validity testing of a single-item physical activity measure Br J Sports Med 2011 45 203 208 10.1136/bjsm.2009.068395 20484314

[B9] Svensson B Markstrom U Bejerholm U Bjorkman T Brunt D Eklund M Hansson L Leufstadius C Gyllensten AL Sandlund M Ostman M Test-retest reliability of two instruments for measuring public attitudes towards persons with mental illness BMC Psychiatry 2011 11 11 10.1186/1471-244X-11-11 21235749 PMC3025948

[B10] Varni JW Limbers CA Neighbors K Schulz K Lieu JE Heffer RW Tuzinkiewicz K Mangione-Smith R Zimmerman JJ Alonso EM The PedsQL Infant Scales: feasibility, internal consistency reliability, and validity in healthy and ill infants Qual Life Res 2011 20 45 55 10.1007/s11136-010-9730-5 20730626

[B11] Garratt AM Ruta DA Abdalla MI Buckingham JK Russell IT The SF36 health survey questionnaire: an outcome measure suitable for routine use within the NHS? BMJ 1993 306 1440 1444 10.1136/bmj.306.6890.1440 8518640 PMC1677883

[B12] Palta M Chen HY Kaplan RM Feeny D Cherepanov D Fryback DG Standard Error of Measurement of 5 Health Utility Indexes across the Range of Health for Use in Estimating Reliability and Responsiveness Med Decis Making 2011 31 2 260 9 10.1177/0272989X10380925 20935280 PMC3607511

[B13] Ravens-Sieberer U Wille N Badia X Bonsel G Burstrom K Cavrini G Devlin N Egmar AC Gusi N Herdman M Feasibility, reliability, and validity of the EQ-5D-Y: results from a multinational study Qual Life Res 2010 19 887 897 10.1007/s11136-010-9649-x 20401552 PMC2892614

[B14] Kullgren JT Galbraith AA Hinrichsen MS Miroshnik I Penfold RB Rosenthal MB Landon BE Lieu TA Health Care Use and Decision-Making among Lower-Income Families in High-Deductible Health Plans Arch Intern Med 2010 170 21 1918 25 10.1001/archinternmed.2010.428 21098352 PMC4004054

[B15] Lieu TA Solomon JL Sabin JE Kullgren JT Hinrichsen VL Galbraith AA Consumer Awareness and Strategies Among Families with High-deductible Health Plans J Gen Intern Med 2009 25 249 254 20033623 10.1007/s11606-009-1184-5 PMC2839340

[B16] Kramer MS Feinstein AR Clinical biostatistics. LIV. The biostatistics of concordance Clin Pharmacol Ther 1981 29 111 123 10.1038/clpt.1981.18 7460469

[B17] Cohen J A Coefficient of Agreement for Nominal Scales Educational and Psychological Measurement 1960 20 37 46 10.1177/001316446002000104

[B18] Cohen J Weighted Kappa - Nominal Scale Agreement with provision for scaled disagreement or partial credit Psychological Bulletin 1968 70 213 19673146 10.1037/h0026256

[B19] Landis JR Koch GG The measurement of observer agreement for categorical data Biometrics 1977 33 159 174 10.2307/2529310 843571

[B20] Maclure M Willett WC Misinterpretation and misuse of the kappa statistic Am J Epidemiol 1987 126 161 169 3300279 10.1093/aje/126.2.161

[B21] Brener ND Collins JL Kann L Warren CW Williams BI Reliability of the Youth Risk Behavior Survey Questionnaire American Journal of Epidemiology 1995 141 575 580 7900725 10.1093/oxfordjournals.aje.a117473

[B22] Spitznagel EL Helzer JE A proposed solution to the base rate problem in the kappa statistic Arch Gen Psychiatry 1985 42 725 728 4015315 10.1001/archpsyc.1985.01790300093012

[B23] Thompson WD Walter SD A reappraisal of the kappa coefficient J Clin Epidemiol 1988 41 949 958 10.1016/0895-4356(88)90031-5 3057117

[B24] Thompson WD Walter SD Kappa and the concept of independent errors J Clin Epidemiol 1988 41 969 970 10.1016/0895-4356(88)90033-9 3193140

[B25] Adejumo AO Heumann C Toutenburg H A review of agreement measure as a subset of association measure between raters Sonderforschungsberelch 386, Paper 385 2004 http://epub.ub.uni-muenchen.de/1755/1/paper_385.pdf

[B26] Brenner H Kliebsch U Dependence of weighted kappa coefficients on the number of categories Epidemiology 1996 7 199 202 10.1097/00001648-199603000-00016 8834562

[B27] Rigby AS Statistical methods in epidemiology. v. Towards an understanding of the kappa coefficient Disabil Rehabil 2000 22 339 344 10.1080/096382800296575 10896093

[B28] Sim J Wright CC The kappa statistic in reliability studies: use, interpretation, and sample size requirements Phys Ther 2005 85 257 268 15733050

[B29] Rosenbaum JE Truth or consequences: the intertemporal consistency of adolescent self-report on the Youth Risk Behavior Survey Am J Epidemiol 2009 169 1388 1397 10.1093/aje/kwp049 19363096 PMC2727247

[B30] Lee SY Poon WY Maximum-Likelihood-Estimation of Polyserial Correlations Psychometrika 1986 51 113 121 10.1007/BF02294004

[B31] Poon WY Lee SY Maximum-Likelihood-Estimation of Multivariate Polyserial and Polychoric Correlation-Coefficients Psychometrika 1987 52 409 430 10.1007/BF02294364

[B32] Banerjee M Capozzoli M McSweeney L Sinha D Beyond kappa: A review of interrater agreement measures Canadian Journal of Statistics 1999 27 3 23 10.2307/3315487

[B33] Agresti A Categorical Data Analysis, Second Edition 2002 Hoboken, New Jersey: Wiley

[B34] McNemar Q Note on the sampling error of the difference between correlated proportions or percentages Psychometrika 1947 12 153 157 10.1007/BF02295996 20254758

[B35] SAS Institute Inc SAS OnlineDoc, Version 9.2 2002 Cary, NC: SAS Institute Inc http://support.sas.com/software/index.html

[B36] Grant BF Dawson DA Stinson FS Chou PS Kay W Pickering R The Alcohol Use Disorder and Associated Disabilities Interview Schedule-IV (AUDADIS-IV): reliability of alcohol consumption, tobacco use, family history of depression and psychiatric diagnostic modules in a general population sample Drug Alcohol Depend 2003 71 7 16 10.1016/S0376-8716(03)00070-X 12821201

[B37] Koziol-McLain J Brand D Morgan D Leff M Lowenstein SR Measuring injury risk factors: question reliability in a statewide sample Inj Prev 2000 6 148 150 10.1136/ip.6.2.148 10875674 PMC1730612

[B38] Nock MK Holmberg EB Photos VI Michel BD Self-Injurious Thoughts and Behaviors Interview: development, reliability, and validity in an adolescent sample Psychol Assess 2007 19 309 317 17845122 10.1037/1040-3590.19.3.309

[B39] Payton ME Greenstone MH Schenker N Overlapping confidence intervals or standard error intervals: what do they mean in terms of statistical significance? J Insect Sci 2003 3 34 15841249 10.1093/jis/3.1.34 PMC524673

[B40] Payton ME Miller AE Raun WR Testing statistical hypotheses using standard error bars and confidence intervals Commun Soil Sci Plant Anal 2000 31 547 551 10.1080/00103620009370458

